# High performance of high-temperature-superconducting MPD thrusters: analytical MHD modeling and experimental demonstration

**DOI:** 10.1093/nsr/nwaf589

**Published:** 2025-12-30

**Authors:** Jinxing Zheng, Yifan Du, Hammad Aftab, Haiyang Liu, Ming Li, Lei Zhu, Yudong Lu, Maolin Ke, Ming Zhu, Juan Wu, Bofan Li

**Affiliations:** Institute of Plasma Physics, Hefei Institutes of Physical Sciences, Chinese Academy of Sciences, Hefei 230031, China; Institute of Plasma Physics, Hefei Institutes of Physical Sciences, Chinese Academy of Sciences, Hefei 230031, China; Scinece Island Branch, Graduate School of USTC, University of Science and Technology of China, Hefei 230026, China; Institute of Plasma Physics, Hefei Institutes of Physical Sciences, Chinese Academy of Sciences, Hefei 230031, China; Scinece Island Branch, Graduate School of USTC, University of Science and Technology of China, Hefei 230026, China; Institute of Plasma Physics, Hefei Institutes of Physical Sciences, Chinese Academy of Sciences, Hefei 230031, China; Institute of Plasma Physics, Hefei Institutes of Physical Sciences, Chinese Academy of Sciences, Hefei 230031, China; Institute of Plasma Physics, Hefei Institutes of Physical Sciences, Chinese Academy of Sciences, Hefei 230031, China; Institute of Plasma Physics, Hefei Institutes of Physical Sciences, Chinese Academy of Sciences, Hefei 230031, China; Institute of Plasma Physics, Hefei Institutes of Physical Sciences, Chinese Academy of Sciences, Hefei 230031, China; Scinece Island Branch, Graduate School of USTC, University of Science and Technology of China, Hefei 230026, China; Institute of Plasma Physics, Hefei Institutes of Physical Sciences, Chinese Academy of Sciences, Hefei 230031, China; Institute of Plasma Physics, Hefei Institutes of Physical Sciences, Chinese Academy of Sciences, Hefei 230031, China; Scinece Island Branch, Graduate School of USTC, University of Science and Technology of China, Hefei 230026, China; Institute of Plasma Physics, Hefei Institutes of Physical Sciences, Chinese Academy of Sciences, Hefei 230031, China; Scinece Island Branch, Graduate School of USTC, University of Science and Technology of China, Hefei 230026, China

**Keywords:** AF-MPD thruster, high-temperature-superconducting MPDT, plasma confinement

## Abstract

The integration of high-temperature superconductors into electric propulsion systems, particularly applied-field magnetoplasmadynamic thrusters (AF-MPDTs), has recently garnered significant attention. However, research on low-power, high-temperature-superconducting (HTS)-based MPDTs, which are crucial for small satellites and CubeSats, remains limited. The increasing demand for compact, high-efficiency propulsion in low Earth orbit underscores the need for scalable HTS-AF-MPDT systems operating below 15 kW. Despite this, challenges such as the lack of detailed theoretical models, limited plasma diagnostics and excessive Joule heating in conventional copper magnets persist. In this work, using a downscaled version of a 25 kW HTS-based AF-MPDT, we address these limitations by developing and experimentally validating a theoretical MHD-based plasma-acceleration model for an AF-MPDT equipped with a conduction-cooled HTS magnet. The system achieves a specific impulse of 3265 s at an input power of 12 kW, more than eight times higher than traditional chemical propulsion, alongside a thrust of 320 mN and an efficiency of 25% at sub-12 kW. The HTS magnet reduces magnetic power consumption from 285 kW to under 1 kW and lowers magnet mass from 220 to 60 kg, enabling substantial improvements in system miniaturization and efficiency. These results represent the first reported demonstration of a 12 kW HTS AF-MPDT, bridging theoretical predictions with experimental outcomes and laying the groundwork for in-orbit demonstration of high-performance propulsion for small satellites.

## Teaser

Chinese scientists have created a lightweight superconducting thruster that cuts power use drastically, making small satellites much more capable for deep space missions.

## INTRODUCTION

Deep-space exploration plays a pivotal role in advancing astronomical research. Propulsion systems for such missions demand high thrust and specific impulse. Electric propulsion (EP) technology, which utilizes electrical energy to accelerate propellant and generate thrust, has become a prominent alternative to traditional chemical propulsion [1]. Unlike chemical propulsion, which relies on the rapid release of stored chemical energy, EP systems efficiently ionize and accelerate controlled amounts of propellant, making them highly power efficient [2].

Electric propulsion has been successfully demonstrated in several deep-space and interplanetary missions. These include the European Space Agency’s SMART-1 lunar orbiter [3], NASA’s Dawn mission to the protoplanets Vesta and Ceres [4], and the Japan Aerospace Exploration Agency’s Hayabusa1 [5] and Hayabusa2 [6] asteroid sample-return missions from Itokawa and Ryugu, respectively.

Electric propulsion systems can be broadly categorized into electrostatic and electromagnetic types, based on their acceleration principles. Among these, the applied-field magnetoplasmadynamic thruster (AF-MPDT) stands out for its theoretical capability to deliver a specific impulse as high as 11 000 s, significantly higher than that of xenon-based ion drives (3500 s) and chemical rockets (455 s) [7]. The AF-MPDT can utilize a variety of propellants, such as xenon, argon, hydrogen, lithium and hydrazine. Unlike ion drives (e.g. Hall thrusters or gridded ion thrusters), which typically generate low thrust [8], the AF-MPDT is theoretically capable of producing thrust levels of up to 200 N. However, such performance requires tens of megawatts of power. In practice, even at input powers of 150 kW, reported thrust levels remain around 4 N [9].

Substantial researches have been conducted to improve the performance and efficiency of AF-MPDTs. High-power AF-MPDTs (100–150 kW, or even megawatt class) demonstrate impressive performance but suffer from substantial energy losses; only a fraction of the input power is converted into thrust. Traditional copper electromagnets are the primary consumers of energy and contributors to system mass. For example, the SX3 thruster prototype from the University of Stuttgart, with a mass of just 13 kg, incorporates a 150 kg copper electro-magnet consuming 285 kW of power [10]. Current space-based power systems (e.g. solar arrays) provide limited output (1400 W/m$^{2}$ at Earth’s orbit), and even large $10\times 10$ m$^2$ arrays can barely supply 56 kW under ideal conditions [11]. Thus, powering a 100 kW thruster would require at least two such arrays, an impractical solution due to mass and space constraints. Additional inefficiencies are attributed to electrical power losses due to Joule heating in copper coils [12].

**Figure 1.**
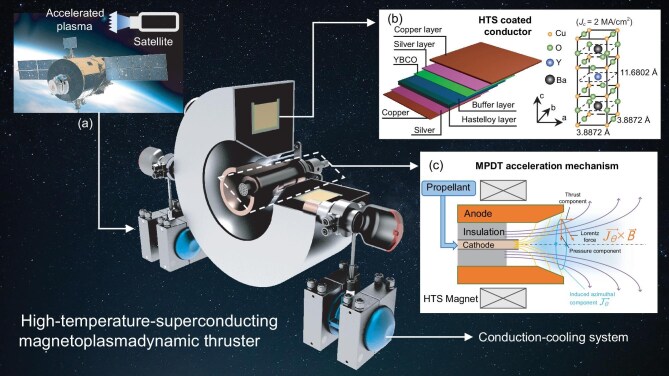
The high-temperature-superconducting applied-field MPDT: (a) schematic of the basic setup of a high-temperature-superconducting applied-field MPDT with central cathode, anode and HTS magnet; (b) multilayer composite structure of high-temperature-superconducting (YBCO) tape; (c) schematic of plasma acceleration in an applied-field MPDT.

These challenges have motivated the development of low-power AF-MPDTs integrating superconducting magnets to replace copper coils. For instance, Zheng *et al.* [9] demonstrated a 150 kW AF-MPDT employing low-temperature superconductors (LTSs), achieving 4 N thrust, a specific impulse of more than 5000-s and an efficiency of 76%. Cathode erosion was minimized owing to the uniform magnetic field generated by the superconducting magnet, which helped direct plasma away from the cathode. However, LTS systems require cryogenic cooling to extremely low temperatures, introducing complexity and mass that hinder practical spacecraft integration. To address this, recent research has focused on high-temperature superconductors, such as YBCO, which remain superconducting at 77 K [13]. The schematic of the multilayer composite structure of the high-temperature-superconducting (HTS) tape is depicted in Fig. 1b; it consists of a copper layer as a stabilizer, a Hastelloy layer, a YBCO layer, a silver layer and a buffer layer. Voronov *et al.* [14], in collaboration with SUPER-Ox, developed a 25–30 kW HTS MPDT, achieving a magnetic field of 1 T, a 300% increase in thrust and a 700% improvement in specific impulse. A maximum specific impulse of 3840 s and an efficiency of 54% were reported. Similar efforts by the University of Stuttgart [15] and Nagoya University [16,17] yielded specific impulses of 1930 s and efficiencies of 26.5% under 1 T HTS magnetic fields. Multiple institutions are currently pursuing HTS-based space propulsion technologies [18,19]. Preliminary experiments using Stirling-cycle conduction cooling have demonstrated central magnetic fields exceeding 0.3 T, with peaks over 0.66 T [20]. HTS magnets have also shown promise in advanced propulsion systems such as VASIMR [21].

Despite recent advancements, HTS-AF-MPDT demonstrations have predominantly operated in the high-power regime (25–150 kW), with applied magnetic fields around 1 T. To date, however, no fully integrated and tested HTS-AF-MPDT system operating below 15 kW has been reported. This limitation presents a critical challenge, particularly in the context of emerging space missions involving small satellites and CubeSats, where low-power propulsion systems are essential due to stringent mass and power constraints. Moreover, the successful launch of the Tiangong space station into low Earth orbit (LEO) in April 2021 underscores the increasing demand for efficient, compact electric propulsion systems to support cargo and crew transport [22]. These developments collectively motivate the present investigation. The primary objective of this work is to design, construct and experimentally evaluate a 10–15 kW HTS AF-MPDT optimized for high performance at low power. Particular emphasis is placed on achieving a high specific impulse, which is a critical parameter for long-duration missions involving satellite constellations and station resupply. Also, HTS-based designs face persistent challenges, including the lack of a comprehensive theoretical framework for plasma acceleration, limited plasma diagnostic analysis and substantial engineering barriers that complicate the realization of a fully integrated HTS system.

Here, we address these issues and report on the development of a compact HTS AF-MPDT operating at 12 kW (Fig. 1), incorporating a conduction-cooled HTS magnet that enables significant reductions in system mass, power consumption and thermal losses. An analytical plasma-acceleration model was developed to examine the physics of plasma expansion in the applied magnetic nozzle (see Fig. 1c), to assess the influence of magnetic field strength on thruster performance. The model predicts a strong dependence of both thrust and specific impulse on the applied magnetic field strength. Experimental validation was carried out using a calibrated thrust stand and target-force measurement method. Results indicate that the use of an HTS magnet reduces coil power consumption from approximately 285 kW—typical for a conventional copper magnet—to less than 1 kW. Additionally, the magnet mass is decreased from 150 to 50–60 kg while maintaining a field strength of 0.2 T. The thruster achieved a specific impulse of 3265 s at a mass flow rate of only 5 mg/s—over eight times higher than that of traditional chemical propulsion systems—along with substantial improvements in overall efficiency at 12 kW input power. These findings confirm the viability of HTS-based AF-MPDTs as compact, high-efficiency propulsion solutions for future low-power electric spacecraft. The successful demonstration at 12 kW represents a crucial step towards the development of scalable HTS-based thruster systems optimized for small-satellite platforms and next-generation exploratory missions.

By combining high specific impulse, reduced system mass and drastically lower magnetic power requirements, the present system addresses key limitations of conventional electric propulsion at low power.

## RESULTS

The results obtained from both analytical and experimental studies are presented here. The axial velocity $v_z$ in the expansion region, obtained by solving the MHD equations, as described in the Methods section below, is given by


(1) \begin{eqnarray*}
v_z = \frac{r^2}{6\mu } \frac{\partial }{\partial z} \bigg ( p + \frac{B_r^2}{\mu _0} \bigg ) + A \ln (r) + B.
\end{eqnarray*}

This equation contains two unknowns, *A* and *B*, which can be determined using the boundary conditions of our experimental HTS-AF-MPDT setup.

### Boundary conditions

**Figure 2.**
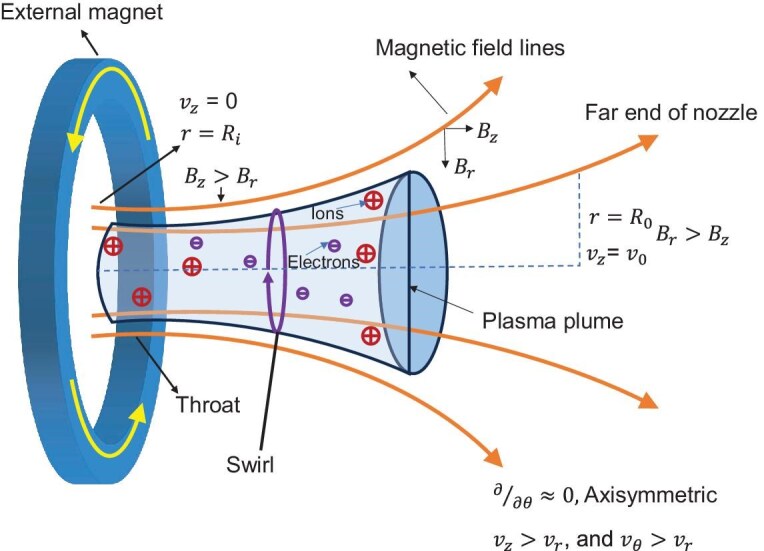
Boundary conditions at the entrance and far end of the diverging magnetic nozzle, set by the high-temperature-superconducting magnet, assuming axisymmetric geometry with $\partial / \partial \theta = 0$, $r = R_i$ at the entrance, and $r = R_0$ at the far end.


Assuming axisymmetric, steady-state flow and employing the nozzle boundary conditions shown in Fig. 2, the conditions can be expressed as follows.

• At the throat entrance ($r = R_i$), the velocity is zero: $v_z = 0$. The nozzle entrance is defined as the interface where the plasma exits the discharge region and enters the nozzle.

• At the nozzle exit ($r = R_0$), the velocity is $v_0$: $v_z = v_0$.

These conditions are expressed as

(2) \begin{eqnarray*}
v_z = \left\lbrace \begin{array}{@{}l@{\quad }l@{}}0 &\quad \text{at } r = R_i,\\
v_0 &\quad \text{at } r = R_0. \end{array}\right.
\end{eqnarray*}

Applying these boundary conditions to Equation (1) yields


(3) \begin{eqnarray*}
\frac{R_i^2}{6\mu } \frac{\partial }{\partial z} \bigg ( p + \frac{B_r^2}{\mu _0} \bigg ) + A \ln (R_i) + B = 0,
\end{eqnarray*}

(4) \begin{eqnarray*}
\frac{R_0^2}{6\mu } \frac{\partial }{\partial z} \bigg ( p + \frac{B_r^2}{\mu _0} \bigg ) + A \ln (R_0) + B = v_0,
\end{eqnarray*}

(5) \begin{eqnarray*}
\frac{R_i^2 - R_0^2}{6\mu } \frac{\partial }{\partial z} \bigg ( p + \frac{B_r^2}{\mu _0} \bigg ) + v_0 = A \ln \bigg (\frac{R_0}{R_i}\bigg ).
\end{eqnarray*}

Here, *p* is the pressure, $B_r$ is the radial magnetic field, $\mu$ is the viscosity, $v_0$ is the velocity at the far end of the magnetic nozzle and $\mu _0$ is the permeability of free space. To avoid lengthy expressions, the values of *A* and *B* obtained by solving Equations (3–5) are provided in the online supplementary material.

The velocity at the far end of the nozzle is obtained as


(6) \begin{eqnarray*}
v_0 = v_{\rm {Hall}} + v_p,
\end{eqnarray*}

where


(7) \begin{eqnarray*}
v_{\rm {Hall}} = \frac{k_1}{\mu _0 (1 - \ln (R_0 / R_i))} \frac{\partial }{\partial z} \left( B_r^2 \right),
\end{eqnarray*}

(8) \begin{eqnarray*}
v_p = \frac{k_1}{1 - \ln (R_0 / R_i)} \frac{\partial }{\partial z} (p).
\end{eqnarray*}

Here, $v_p$ is basically the velocity contribution due to the pressure gradient, and $v_{\rm {Hall}}$ is the velocity contribution due to the magnetic field effect. The expressions $k_1$ and $k_2$ are lengthy and depend on geometric factors. Their detailed forms are provided in the online supplementary material.

Substituting Equations (6–8) into the expressions for *A* and *B* in the online supplementary material (Equations (A1) and (A2)), and then inserting the resulting values into Equation (1) yields the final expression for the axial velocity:


(9) \begin{eqnarray*}
v_z = k_3 \frac{\partial }{\partial z} (p) + v_p k_2 + \frac{k_3}{\mu _0} \frac{\partial }{\partial z} \left(B_r^2\right) + v_{\rm {Hall}} k_2.
\end{eqnarray*}

Similarly, for the swirl component of the acceleration, the *r*-component of momentum equation (29) below can be simplified to obtain the azimuthal velocity as follows:


(10) \begin{eqnarray*}
v_\theta = \frac{r}{\sqrt{2\rho }} \frac{\partial }{\partial z} \Bigg ( \sqrt{p + \frac{B_r^2}{\mu _0}} \Bigg ).
\end{eqnarray*}

The current density component $J_z B_r$ obtained for the $\theta$-component of momentum equation (30) below is given by


(11) \begin{eqnarray*}
J_z B_r = -\bigg [ \frac{1}{r^2} \frac{\partial }{\partial r} \bigg \lbrace r \frac{\partial }{\partial r} \bigg ( \frac{v_\theta }{r} \bigg ) \bigg \rbrace \bigg ].
\end{eqnarray*}

The swirl component of the thrust, thus obtained, is given in Equation (A6) in the online supplementary material.

### Total thrust components

The total plasma acceleration of an applied-field MPDT generally comprises four components:


(12) \begin{eqnarray*}
T_{\rm {total}} = T_{\rm {Hall}} + T_{\rm {swirl}} + T_{\rm {gas,dynamics}} + T_{\rm {Self}}.
\end{eqnarray*}

The geometry of the thruster and the input power determine which component dominates. The Hall and swirl accelerations can be written as


(13) \begin{eqnarray*}
T_{\rm {Hall}} = \frac{k_3}{\mu _0} \displaystyle\frac{\partial }{\partial z} \left(B_r^2\right) + v_{\rm {Hall}} k_2,
\end{eqnarray*}

(14) \begin{eqnarray*}
T_{\rm {gas,dynamics}} = k_3 \frac{\partial p}{\partial z} + k_2\, v_p,
\end{eqnarray*}

where $T_{\rm {swirl}} = J_z B_r$, which can be written as


(15) \begin{eqnarray*}
T_{\rm {swirl}} = - \bigg [ \frac{1}{r^2} \frac{\partial }{\partial r} \bigg \lbrace r \frac{\partial }{\partial r} \bigg ( \frac{v_\theta }{r} \bigg ) \bigg \rbrace \bigg ].
\end{eqnarray*}

Typically, the axial velocity in the expansion region differs from the exhaust velocity. As noted by Sasoh [23], it is observed to be about half the exhaust velocity, even when swirl is present: $v_z = \frac{1}{2} v_{\rm {exhaust}}$. Assuming axisymmetric flow with magnetic fields only in the *r* and *z* directions ($\mathbf {B} = B_r \hat{r} + B_z \hat{z}$), the self-contribution is given by


(16) \begin{eqnarray*}
T_{\rm {self}} = \frac{B_\theta }{\pi r z_1^2} (z_0 - z_1),
\end{eqnarray*}

where $B_\theta = {\mu _0 I_D}/{2 \pi r}$. Thus, Equation (16) can be further expressed as


(17) \begin{eqnarray*}
F_{\rm {self}} = \int _0^{z_1} \int _0^{2\pi } \int _{r_c}^{r_a} \frac{B_\theta }{\pi r z_1^2} (z_0 - z_1) r \, dr \, d\theta \, dz.
\end{eqnarray*}

Here, $I_D$ denotes the discharge current, $r_c$ and $r_a$ represent the cathode and anode radii, respectively, and *z* is the axial coordinate. Performing the integration then yields


(18) \begin{eqnarray*}
F_{\rm {self}} = \frac{B_\theta r I_D}{2} \bigg ( 1 - \frac{z_0}{z_1} \bigg ) \ln \bigg ( \frac{r_a}{r_c} \bigg ),
\end{eqnarray*}

which vanishes for $B_{\theta } = 0$, resulting in $F_{\rm {self}} = 0$. In this study, multiplying the velocity (Equation (9)) by the mass flow rate on both sides gives the total force, expressed as the sum of two components: (i) the Hall term, arising from the interaction of azimuthal currents with the radial and axial magnetic fields; and (ii) the pressure-dynamic (or gas-dynamic) term. The third contribution, the swirl term, is treated separately from Equation (15), as it originates from the interaction of radial and axial currents with the magnetic field, where the resulting plasma gyration is redirected into axial thrust by the magnetic nozzle. The total thrust is thus obtained as the sum of these three components:


(19) \begin{eqnarray*}
F_{\rm {total}} = F_{\rm {Hall}} + F_{\rm {gas,dynamics}} + F_{\rm {swirl}}.
\end{eqnarray*}

The pressure contribution depends on the rate of change of pressure along the axial direction. Since the pressure usually varies mainly along the radial direction, this contribution is very small and can therefore be neglected. The thrust force contribution can be written as


(20) \begin{eqnarray*}
F_{\rm {gas,dynamics}} = \dot{m}\, T_{\rm {gas,dynamics}},
\end{eqnarray*}

where $T_{\rm {gas.dynamic}}$ is given in Equation (14). Since Equation (20) gives a negligible contribution due to pressure variation along the *r* direction only, we are left with two components: swirl and Hall. The Hall contribution can be written as


(21) \begin{eqnarray*}
F_{\rm {Hall}} = \left[ \displaystyle\frac{\dot{m} k_3}{\mu _0} + \displaystyle\frac{\dot{m} k_1 k_2}{\mu _0 ( 1 - \ln (R_0 / R_i) )} \right] \displaystyle\frac{\partial }{\partial z} \left( B_r^2 \right).\\
\end{eqnarray*}

The corresponding thrust relations, obtained by integrating the swirl expressions, can be written as


(22) \begin{eqnarray*}
F_{\rm {total}} = F_{\rm {Hall}} + F_{\rm {swirl}}.
\end{eqnarray*}

We denote by $\dot{m}$ the mass flow that is constant in this case, and the thrust components are $F_{\rm {swirl}}=\int\!\int _{r,z} J_z B_r\, dr\, dz=\int\!\int _{r,z} T_{\rm {swirl}}\, dr\, dz$. For brevity, the detailed expression for $F_{\rm {swirl}}$ is given in the online supplementary material, where $T_{\rm {swirl}}$ is evaluated along the radial and axial directions to obtain the final relation for the swirl contribution.

In our thruster design, the radial magnetic field $B_r$ is related to the magnetic field at the throat and the divergence angle of the nozzle [24]:


(23) \begin{eqnarray*}
B_r^2 = B_{\rm {th}}^2 \tan ^2(\theta _D).
\end{eqnarray*}

Here, $\tan (\theta _D)={C L_c}/{(R_{\rm {inner}}+R_{\rm {outer}})}$, the length of the magnetic nozzle is given by $L_m=R_{\rm {in}}\sqrt{ \left(\frac{B_{\rm {th}}}{B_{\rm {exit}}} \right)^{2/3}-1}$and ${\partial }(B_r^2)/{\partial z}\approx {B_r^2}/{L_m}$ (for a slowly diverging nozzle). The exit magnetic field can be estimated using Ampère’s law as $B_{\rm {exit}} = 0.0016\ \mathrm{T}$ for a discharge current $I_d = 160\ \mathrm{A}$ and exit radius $r_{\rm {exit}} = 20\ \mathrm{mm}$, which defines the area of the exit plane. Thus, after solving the integrals in Equation (22) and Equation (A6) in the online supplementary material for the swirl component, the total thrust relation obtained is given by Equation (A7) in the online supplementary material.

The relation in Equation (A7) in the online supplementary material is plotted for different magnetic field strengths. Here, $B_{\rm {th}}$ is the axial magnetic field at the throat, which is the same as $B_{\rm {th}} = B_{\rm {applied}}$. The parameter *C* is the fitting parameter representing the slope of the magnetic field lines and any deviations caused by simplifications.

We observe that an increase in the magnetic field strength enhances the thrust of the AF-MPDT. Also, $F_{\rm {swirl}}$ has an inverse dependence on the density/mass flow rate. Therefore, at low mass flow rates, $F_{\rm {swirl}}$ is higher than $F_{\rm {Hall}}$.

### Analytical thrust calculation

**Figure 3.**
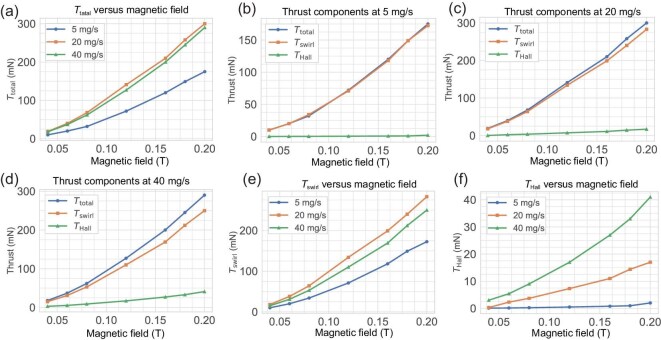
Calculated thrust from the analytical model as a function of the applied magnetic field for mass flow rates of 5, 20 and 40 mg/s. (a) Thrust versus the applied magnetic field for 5, 20 and 40 mg/s, respectively. (b–d) Different components of thrust versus the applied field at 5 mg/s, 20 mg/s and 40 mg/s, respectively. (e) Component $F_{\rm {swirl}}$ versus the applied magnetic field at 5, 20 and 40 mg/s, respectively. (f) Component $F_{\rm {Hall}}$ versus the applied magnetic field at 5, 20 and 40 mg/s, respectively.

The analytical model provides intriguing insights. In Fig. 3a, it is evident that an increase in the applied magnetic field enhances the thrust generated by the thruster. The maximum thrust, approximately 300 mN, is achieved at a mass flow rate of 20 mg/s. At 40 mg/s, the peak thrust is 290 mN, while at 5 mg/s, a slightly reduced thrust of 170 mN is attained. The model establishes a clear correlation between increased thrust and the applied magnetic field, aligning with previous analytical models proposed by Fradkin *et al.* [25], Tikhonov *et al.* [26] and Hedrich *et al.* [27]. Both $F_{\rm {swirl}}$ and $F_{\rm {Hall}}$ exhibit dependence on the applied magnetic field.

In Fig. 3b, $F_{\rm {swirl}}$, $F_{\rm {Hall}}$ and $F_{\rm {total}}$ for different field strengths at 5 mg/s are illustrated. Notably, $F_{\rm {total}}$ and $F_{\rm {swirl}}$ closely match each other, whereas $F_{\rm {Hall}}$ is considerably lower and diverges even at low magnetic field strengths.

Figure 3c showcases $F_{\rm {swirl}}$, $F_{\rm {Hall}}$ and $F_{\rm {total}}$ at a mass flow rate of 20 mg/s. This indicates that $F_{\rm {Hall}}$ at 20 mg/s is more potent than $F_{\rm {Hall}}$ at 5 mg/s. However, $F_{\rm {total}}$ and $F_{\rm {swirl}}$ remain closely aligned, reaffirming $F_{\rm {swirl}}$ as the primary thrust component at 20 mg/s, though a discernible difference between $F_{\rm {total}}$ and $F_{\rm {swirl}}$ becomes visible at higher magnetic fields above 0.12 T.

Inspecting the thrust at a mass flow rate of 40 mg/s in Fig. 3d, the discrepancy between $F_{\rm {total}}$ and $F_{\rm {swirl}}$ has further increased, yet $F_{\rm {swirl}}$ remains the primary thrust component. The peak value of $F_{\rm {Hall}}$ has escalated to 40 mN, contrasting with the earlier values of 2 mN at 5 mg/s and 17 mN at 20 mg/s. This indicates that, at higher mass flow rates, the $F_{\rm {Hall}}$ component intensifies, while the $F_{\rm {swirl}}$ component diminishes. As the mass flow rate decreases, the $F_{\rm {swirl}}$ component intensifies due to its inverse dependency on the mass flow rate. In Fig. 3e, three $F_{\rm {swirl}}$ components at different mass flow rates are revealed. As the magnetic field increases, we observe that $F_{\rm {swirl}}$ at 20 and 40 mg/s surpasses that at 5 mg/s. A decrease in the mass flow rate increases the thrust, but at the same time, the dependency on the magnetic field makes the relationship complex. Because of the highest swirl at 20 mg/s, the total thrust at 20 mg/s is higher compared to the others.

In Fig. 3f, the three $F_{\rm {Hall}}$ components at different mass flow rates highlight that $F_{\rm {Hall}}$ for 40 mg/s exceeds those at 20 and 5 mg/s, unmistakably indicating that increasing the mass flow rate enhances $F_{\rm {Hall}}$. Given that $F_{\rm {Hall}}$ exhibits a linear dependence on the mass flow rate—and considering the relatively low values of 40, 20 and 5 mg/s—$F_{\rm {Hall}}$ remains low. However, since $F_{\rm {swirl}}$ has an inverse-square-root dependence on the mass flow rate, it remains more prominent than $F_{\rm {Hall}}$. At 40 mg/s, $F_{\rm {Hall}}$ is 41 mN, significantly higher compared to 2 and 17 mN at 5 and 20mg/s, respectively, but still not enough to surpass $F_{\rm {swirl}}$.

Component $F_{\rm {swirl}}$ has an inverse dependence on the mass flow rate, whereas $F_{\rm {Hall}}$ has a linear dependence, which makes the total thrust relationship very complex. Moreover, the linear and quadratic dependencies on the magnetic field further complicate the relation, resulting in 20 mg/s being the mass flow rate at which the highest thrust is achieved. The mass flow rate also depends on the expansion area of the nozzle.

In conclusion, increasing the mass flow rate strengthens $F_{\rm {Hall}}$ while concurrently diminishing $F_{\rm {swirl}}$, but decreasing the mass flow rate strengthens $F_{\rm {swirl}}$, with the magnetic field playing a very crucial role. Our analytical findings substantiate that the dependence of thrust on the applied field in our case is attributed to the dominant $F_{\rm {swirl}}$ component. When the mass flow rate reaches a sufficiently high level, $F_{\rm {Hall}}$ becomes a more significant contributor than $F_{\rm {swirl}}$. However, since the mass flow rates under consideration are low, $F_{\rm {swirl}}$ remains high in our case.

To validate our theoretical results, a meticulous experiment was conducted using a 25 kW setup downscaled to a 10–15 kW HTS AF-MPDT. A detailed comparison and presentation of the results will be discussed in the forthcoming sections.

### Experimental results and comparison

The detailed experiment was performed, and the results obtained were compared with the theoretical model, showing very strong agreement at higher magnetic field strengths and low mass flow rates. However, at high mass flow rates and low magnetic field strengths, due to the isothermal approximation, the errors are larger. Thus, the present model captures the behavior of thrust more accurately at low mass flow rates and higher magnetic fields. Overall, the behavior of high thrust at 20 mg/s is consistent with the theoretical model.

## Cooling of the HTS magnet

**Figure 4.**
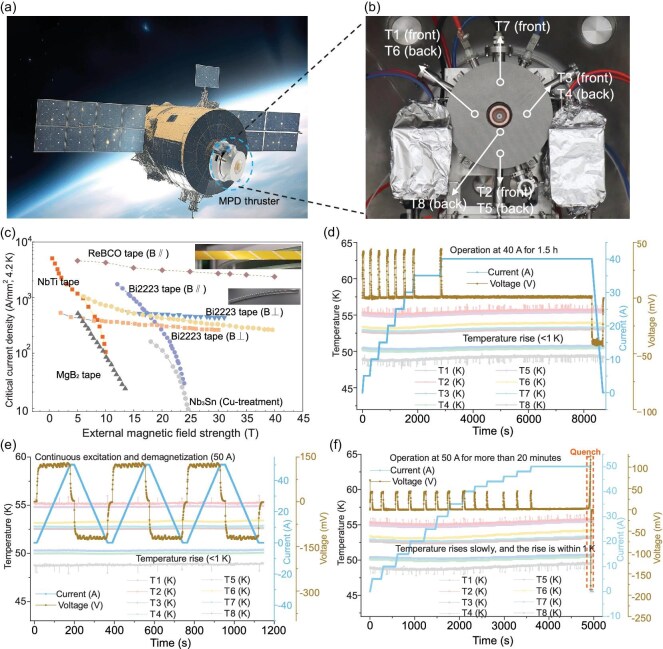
Illustrations of superconducting MPDTs. (a) A photograph of a satellite using electric propulsion engines. (b) Experimental setup of an HTS-enhanced MPDT integrated with a Stirling conduction-cooling system, showing the HTS magnet installed in the setup. (c) The current state of superconducting tapes used in MPDT magnets, typically employing NbTi and Nb$_3$Sn tapes at low temperatures (4.2 K) and YBCO tapes at higher temperatures (4.2–77 K) [28]. Panels (d–f) show the operating results of the high-temperature-superconducting magnet used in this study, with T1–T8 indicating the positions of the temperature sensors (eight PT100 thermometers) on the single pancake coil.

First, the cooling results of the high-temperature superconductors used as an external magnet are presented here. Panels (a) and (b) of Fig. 4 show the experimental setup with high-temperature superconductors integrated. The setup provides an internal view of the thruster with HTS magnet, cathode and anode. The view represents a sub-15 kW HTS MPDT.

Labels T1–T8 indicate the positions of the temperature sensors (eight PT100 thermometers) on the single pancake coil. Positions T1, T2, T3 and T7 correspond to the frontal temperature sensors, while T4, T5, T6 and T8 correspond to the rear temperature sensors.

To meet the demands of MPDTs for spaceflight applications and achieve a lightweight design with a stable magnetic field, we present a scheme for a HTS-based AF-MPDT incorporating a miniaturized Stirling conduction-cooling system. High-temperature superconductivity, a crucial aspect of this proposed solution, refers to the phenomenon where materials exhibit superconducting properties at relatively higher temperatures, typically in the liquid nitrogen temperature zone.

The HTS coils in our setup operate reliably at temperatures between 50–60 K, demonstrating steady-state performance at 40 A. The magnet used in this study is similar to that described in [20], consisting of four double-pancake coils, each with $160 \times 2$ turns, a radial thickness of approximately 75 mm and an outer radius of 117.5 mm. The magnet is wound with 4-mm-wide YBCO HTS tape and maintains stable operation around 60 K. The maximum magnetic field generated by the superconducting magnet does not exceed 1 T, corresponding to a critical current of 120 A. Since the actual operating current never exceeds 60 A, the system operates with a safety margin greater than 50%. To protect the superconducting coils, a heat-resistant mica sheet is placed at the nozzle exit to shield against residual plasma impact. Extensive stability checks were carried out during the design phase, and analysis under the maximum current of 60 A shows a stability margin exceeding 1000 mJ/cc—an order of magnitude higher than typical low-temperature-superconducting systems, ensuring robust operation. In addition, a quench protection system enables rapid shutdown in case of disturbances, further guaranteeing magnet safety. With this HTS magnet, we achieved significantly higher performance in thrust, specific impulse and efficiency, particularly at low power levels.

Considering the stress, structural strength and performance factors of the coil, we opted for commercially viable YBCO superconducting tapes [13,19], as depicted in Fig. 4c, and employed conduction cooling instead of large compressor-based conventional Gifford–McMahon (GM) cooling systems using liquid helium or nitrogen. This represents a significant step toward the miniaturization of space-based MPDTs, and integrated testing of the thruster system has been successfully accomplished. This miniaturized HTS magnet system has reduced the complete magnet system size from 150 to 60 kg, including the skeleton. For the simulation of the magnetic field topology of the HTS magnet, see the online supplementary material.

The cooling of the HTS magnet using a conduction system is shown in Fig. 4d–f. Before cooling commenced, the air inside the system was evacuated to a stable vacuum level of about $10^{-3}$ Pa. Cooling was performed with four cryocoolers, which lowered the magnet temperature to the desired level, as monitored by the cooling curve. After 7 h of cooling, the temperature stabilized and the magnet reached thermal equilibrium with its surroundings. Panels (d–f) of Fig. 4 present the performance of the conduction-cooled HTS magnet. As current flowed through the coil, resistive heating was dissipated by the cooling system. In Fig. 4d, the current was ramped from 0–40 A at 0.1 A/s in 5 A steps, held at 40 A for 1.5 h, and then ramped down at the same rate. No quench occurred, and the temperature rise remained within 1 K, confirming effective heat removal during current variations. In Fig. 4e, the current was ramped up to 50 A (above the 40 A design) and held for 20 min before quench occurred, with temperature fluctuations still within 1 K. Figure 4f shows voltage and temperature responses under continuous AC excitation, demonstrating stable operation without quench. Continuous excitation and demagnetization tests at 50 A confirm the robust electrical performance of the HTS magnet, meeting the required magnetic field of 0.2 T.

### Plasma diagnostic analyses

**Figure 5.**
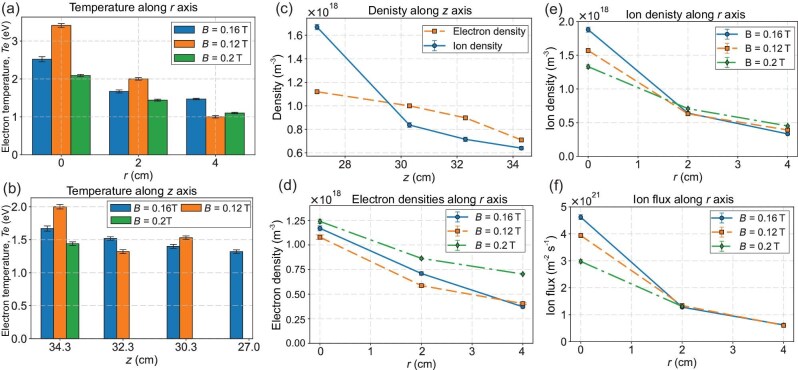
Plasma diagnostic analysis of the plume using a single Langmuir probe. (a and b) Variation of plasma temperature with central magnetic field strength along the radial and axial directions. (c) Electron and ion densities along the axial direction. (d) Radial profiles of the electron density at different magnetic field strengths. (e) Radial profiles of the ion density under varying magnetic field strengths. (f) Ion flux trends along the radial direction at different magnetic field strengths.

**Figure 6.**
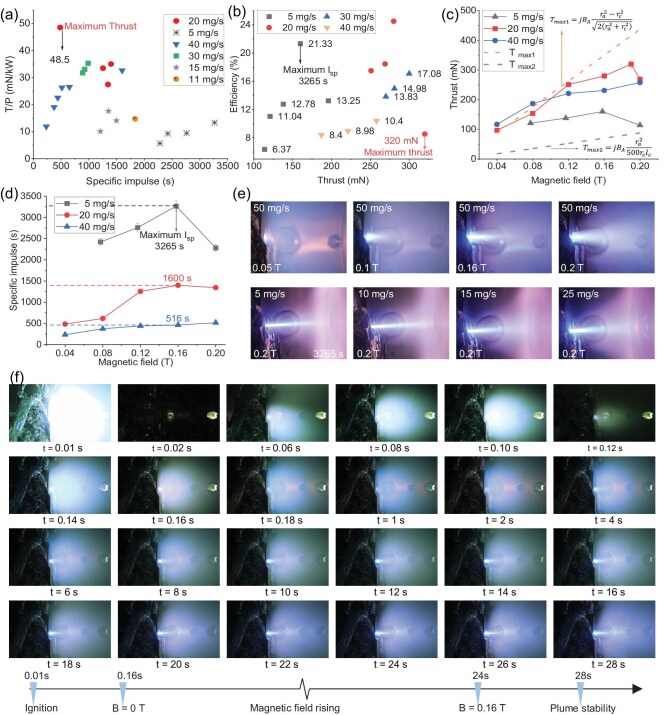
The experimental results of thruster Ignition test and thrust measurement conducted in a large vacuum chamber. (a) Thrust-to-power ratio versus specific impulse for mass flow rates of 5, 20 and 40 mg/s, respectively. (b) System efficiency versus thrust for mass flow rates of 5, 20, 30 and 40 mg/s, respectively. (c) Thrust versus applied magnetic field at 5, 20 and 40 mg/s, respectively. (d) Specific impulse versus applied magnetic field at 5, 20 and 40 mg/s, respectively. (e) Plume images observed under different field strengths (0.05–0.2 T) and mass flow rates (5–25 mg/s). (f) High-speed camera images showing plume stability and acceleration under the applied magnetic field over time.

A single Langmuir probe—referred to as the Hidden Espion probe—is initially employed and mounted on a movable stage to measure plasma parameters, including density, temperature and the electron energy distribution function, at various axial and radial positions under different magnetic field strengths produced by the HTS magnet. Figure 5c shows the axial variation of electron and ion densities at $B = 0.16$ T. Both decrease axially from 27 to 34.3 cm, with maximum values at 27 cm. At 27 cm, the ion density slightly exceeds the electron density before experiencing a sharper downstream drop. Figure 5d highlights the radial variation in electron density across all magnetic field strengths ($B=0.12, 0.16$ and 0.2 T), with the slowest rate of decline observed at $B =0.2$ T. Figure 5e shows a similar radial decrease for the ion density. The slowest decreases in the densities of both ions and electrons at 0.2 T are driven by core confinement and plume expansion, which is also confirmed by the increase in the plume length with applied magnetic field strength in Fig. 6e. The peak ion density ($2 \times 10^{18}\ \mathrm{m}^{-3}$) and highest ion flux ($5 \times 10^{21}\ \mathrm{m}^{-2}\, \mathrm{s}^{-1}$) occur at $B = 0.16\ \mathrm{T}$, as shown in Fig. 5f. Since the highest ion flux is obtained at 0.16 T, we will see shortly that the highest thrust is also achieved at the same magnetic field strength. Plasma temperature decreases radially and axially, peaking at 3.5 eV at $r = 0$ cm and $B = 0.12$ T. At 34.3 cm axially and $r = 2$ cm, the temperature is 2 eV for $B = 0.12$ T and 1.6 eV for $B = 0.16$ T, as depicted in Fig. 5a–b. Thus, plasma diagnostics show that the highest plasma confinement and ion flux are obtained between 0.16 and 0.2 T, while electrons are more energetic at 0.12 T. Since the MPDTs is primarily an ion engine, its highest performance is expected in the 0.16–0.2 T range, as will be discussed shortly.

### Performance improvement

The plume stability and its acceleration with the magnetic field are depicted in Fig. 6f. Initially, at ignition (around $t = 0.18\ \mathrm{s}$), the plume begins to form as the argon gas is ionized into a stable plasma, while the external magnetic field remains off. At $t = 6\ \mathrm{s}$, the external field is activated, leading to a stabilized plume. A fully stable plume is observed between $t = 25\\!-\\!28\ \mathrm{s}$, marking the period during which probe and thrust measurements were initiated.

The magnetic field is limited to 0.2 T (25 A) to ensure safe magnet operation. This precaution is necessary because the HTS tapes are costly and must be operated within their rated current range to prevent degradation, particularly since the temperature in the discharge region of the applied-field MPDT can reach 2–5 eV.

Figure 6a shows the thrust-to-power ratio versus specific impulse. The highest thrust-to-power ratio of $48.5\ \mathrm{mN/kW}$ is obtained at a mass flow rate of $20\ \mathrm{mg/s}$, as depicted in Fig. 6a. Thrust-to-power ratios at various flow rates are presented, with the maximum consistently occurring at $20\ \mathrm{mg/s}$. Figure 6b shows the efficiency as a function of thrust for different mass flow rates. A peak efficiency of 25% is achieved at $20\ \mathrm{mg/s}$ and 12 kW, which is among the highest reported for low-power MPDTs. It is evident that the efficiency increases with thrust.

Figure 6c also shows the thrust obtained in the experimental setup with applied magnetic field. The thrust increases with applied field, although a small decrease is observed at 0.2 T. The thrust is further compared with previously calculated models, revealing that the total thrust at a low mass flow rate of 5 mg/s closely matches the predictions made by Myers *et al.* [29], while, as the flow rate increases, the trend shifts toward Fradkin’s predictions. In the thrust graph (Fig. 6c, T_max1_ represents the theoretical maximum MPDT thrust as deduced by Fradkin *et al.* [25], while T_max2_ is based on the predictions of Myers *et al.* A notable finding of this study is that the highest thrust was observed in the magnetic field range of 0.16–0.18 T. This corresponds precisely to the field strength at which our plasma diagnostics indicated the peak ion flux and ion density, as shown in Fig. 5f. This correlation strongly supports the conclusion that increased ion flux leads directly to higher thrust output, reinforcing the effectiveness of our magnetic field configuration in optimizing performance. Additionally, advancements in thruster design, particularly the integration of an HTS magnet with a Stirling conduction-cooling system, played a pivotal role in achieving a remarkable specific impulse of approximately 3265 s at a modest power input of 12 kW. This value is nearly eight times higher than that of conventional chemical propulsion systems. Such high specific impulse at low power levels is especially well suited for small-satellite and CubeSat missions, where efficiency and compactness are paramount. While the thrust of 320 mN may appear modest, it is sufficient for these platforms, where impulse, rather than force, is often the primary requirement. As shown in Fig. 6d, specific impulses of 1600 and 516 s were also achieved at mass flow rates of 20 and 40 mg/s, respectively, further demonstrating the system’s performance flexibility.

### Comparison with previous low-power AF-MPDTs

The comparison with previous low-power thrusters serves solely to elucidate the advancements made. Our device, the HTS AF-MPDT, achieved an impressive 25% efficiency and a specific impulse of 3265 s, the highest specific impulse achieved thus far in MPDTs at low power ($<$15 kW). Moreover, the thrust of 320 mN is obtained. Most importantly, our device boasts a lightweight design, a stark departure from the heavy copper coils utilized by other thrusters. This lightweight design perfectly addresses the issue of using electric propulsion devices for small satellites and CubeSats. Figure 6e shows stable plume images at different mass flow rates and magnetic field strengths. The plume length increases with increasing magnetic field strength. At low mass flow rates, the plume appears sharper and more collimated, whereas at higher flow rates, the plume becomes visibly thicker and more diffuse. The voltage absorbed by the plasma to generate thrust, as well as the voltage lost to the electrodes and their variation with magnetic field strength, are provided in the online supplementary material. The detailed variation of power with magnetic field strength at different mass flow rates is provided in the online supplementary material and in [13]. Thus, the configuration demonstrating a 12 kW setup, a specific impulse of 3265 s, 320 mN thrust and 25% efficiency represents a promising foundation for further optimization and in-orbit demonstration on small satellites and CubeSats.

**Figure 7.**
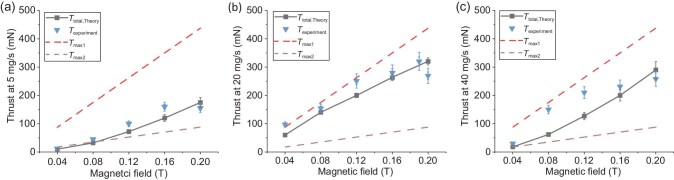
Comparison between theoretical and experimental thrust results. (a–c) The thrust comparison at 5 mg/s, 20 mg/s and 40 mg/s, respectively.

Because of the isothermal approximation used in the theoretical model, the results show some discrepancy as the mass flow rate reaches 40 mg/s, but at 5 and 20 mg/s the results align fairly well with the theoretical model, as depicted in Fig. 7a–c. At 40 mg/s and lower magnetic field strengths, the discrepancy is large, but in the magnetic field strength range of 0.16 to 0.2 T, a remarkable alignment is observed, with peak values closely corresponding to theoretical expectations. This correlation provides compelling evidence for the accuracy of our analytical model. The results were also compared with previous thrust models and show that the thrust lies within the predicted range. T_max1_, T_max2_ represent the previous theoretical models. Table 1 shows a comparison with previous low-power MPDT studies reported in the literature. The 25% efficiency achieved in our 12 kW configuration exceeds previously reported values at similar power levels, mass flow rates and magnetic field strengths. In summary, our study presents a comprehensive exploration of AF-MPDTs, showcasing their capability to deliver a thrust of 320 mN, a specific impulse of 3265 s and an efficiency of 25% at a low power input of less than 15 kW. A consistent trend of thrust versus magnetic field was observed in both cases. Theoretical predictions closely matched experimental findings, particularly evident in the highest thrust achieved at a 2 0mg/s flow rate in the experimental setup, aligning with theoretical expectations. Notably, at high magnetic fields, efficient ionization in the experimental setup led to a precise match between theoretical and propellant flow rates.

**Table 1.** Comparison of applied-field MPDT performances.

	Power	*B* field			Thrust	Flow
Thruster	(kW)	(T)	Magnet	$I_{sp}$ (s)	(mN)	rate
Beihang Uni. [30]	2–11	0–0.13	Cu	1400 (Ar)	296 (Ar)	42 mg/s
Nagoya Uni. [31,32]	0.7–9	0.13–0.26	Cu	588	136	1.5–2.7 mg/s
	0.9–2.8	0.3–1.0	HTS	2050	25–50	14–28 sccm
Waseda Uni. [33]	0.5–1.1	0.1–0.2	Cu	1600 (Ar)	12 (Ar)	100 sccm
				1100 (Ne)	7.2 (Ne)	
				2200 (He)	4.2 (He)	
HTS AF-MPDT (This work)	8–12	0–0.2	HTS	3265 (Ar)	320 (Ar)	5–40 mg/s

## CONCLUSIONS

This work presents the first combined theoretical and experimental investigation of an HTS-based AF-MPDT. We introduce the first theoretical model specifically developed for AF-MPDTs employing HTS magnets. A key result is the demonstration of 320 mN of thrust and a specific impulse of 3265 s, with a measured efficiency of 25% at a power input of just 8 kW, a level readily achievable with standard satellite solar arrays. This specific impulse is approximately eight times greater than that of conventional chemical propulsion and three times higher than typical ion-based systems. The adoption of an HTS magnet addresses long-standing challenges associated with the mass and power consumption of copper coils. The complete HTS system, including cryogenic cooling and structural support, weighs only 60 kg, significantly less than the 150–200 kg required for equivalent copper-based systems. Furthermore, magnetic power requirements were reduced from 285 kW (copper) to below 1 kW (HTS), enabling this high-impulse operation at low power. Our results represent the highest specific impulse reported for any MPDT operating in the low-power regime ($<$15 kW) and affirm the feasibility of compact AF-MPDTs for small-satellite missions. These findings advance the field of electric propulsion, representing a significant step toward the development of MPDTs as a scalable solution for support, cargo and crew transport in future space missions.

## Methods and materials

The 12 kW applied-field MPDT configuration is shown in Fig. 1. The design features a central tungsten cathode with an inner radius of 1 mm and an outer radius of 3 mm, and a conical anode with an outer radius of 40 mm. The magnetic field is generated by an HTS magnet capable of producing a field strength of 0.2 T. The HTS magnet schematic material composition is depicted in Fig. 1. A detailed analytical thrust model was first developed using a magnetohydrodynamic (MHD) framework to describe plasma acceleration. The model’s predictions were then compared with experimental results to validate its accuracy. Figure 1 also presents a schematic of the basic HTS setup.

### Theoretical model

The MHD equations provide the foundational framework for describing electrically conducting fluids, such as those found in AF-MPDTs. These equations couple Maxwell’s equations with the Navier–Stokes equations to capture the intricate interplay between electromagnetic fields and plasma flow. Kubota [34] reported a Knudsen number of approximately 0.02 for AF-MPDTs, suggesting that the continuum assumption may be marginal. However, the presence of a strong external magnetic field justifies the use of a fluid description by effectively mimicking collisional behavior and supporting the maintenance of a Maxwellian distribution. Consistent with this approach, prior simulations of AF-MPDTs have employed MHD models to study plasma behavior [35].

The mass conservation is given by the continuity equation


(24) \begin{eqnarray*}
\nabla \cdot (\rho \mathbf {v}) = 0,
\end{eqnarray*}

where $\rho$ and $\mathbf {v}$ are the density and velocity of the plasma plume, respectively.

The momentum equation is expressed as


(25) \begin{eqnarray*}
\rho (\mathbf {v} \cdot \nabla )\mathbf {v} = \mathbf {F}_v + \nabla \cdot (-P\boldsymbol {\delta } + \mathbf {K}),
\end{eqnarray*}

where *P* is the pressure tensor, $\boldsymbol {\delta }$ is the identity matrix, the volume force


(26) \begin{eqnarray*}
\mathbf {F}_v = \mathbf {J} \times \mathbf {B},
\end{eqnarray*}

and the stress tensor


(27) \begin{eqnarray*}
\mathbf {K} = \mu [ \nabla \mathbf {v} + (\nabla \mathbf {v})^T ] + \kappa \mu (\nabla \cdot \mathbf {v}) \mathbf {I}.
\end{eqnarray*}

Here, $\mu$ denotes the first coefficient of viscosity, and $\kappa$ denotes the second coefficient of viscosity, which represents the shear stresses in the model. Generally, its value is taken as $\kappa = -\frac{2}{3}$.

The Maxwell equations are


(28a) \begin{eqnarray*}
\nabla \cdot \mathbf {E} = -\frac{\rho _q}{\varepsilon _0},
\end{eqnarray*}

(28b) \begin{eqnarray*}
\nabla \times \mathbf {E} = -\frac{\partial \mathbf {B}}{\partial t},
\end{eqnarray*}

(28c) \begin{eqnarray*}
\nabla \cdot \mathbf {B} = 0,
\end{eqnarray*}

(28d) \begin{eqnarray*}
\nabla \times \mathbf {B} = \mu _0 \mathbf {J},
\end{eqnarray*}

where $\mathbf {J}$ is the current density.

Because of the highly nonlinear nature of the MHD equations, obtaining an analytical solution for a complex system like the AF-MPDT is not feasible. However, we employed a technique to derive an approximate analytical solution that effectively describes the acceleration of plasma in the AF-MPDT. Our model operates under the following assumptions.

• Plasma is fully ionized, and the model follows an axisymmetric approach.

• The radial velocity $v_r$ is negligible compared to the axial and azimuthal velocities, and the axial gradient of the azimuthal velocity, $\partial v_\theta / \partial z$, can be taken as zero [35–37].

• In the expansion region, $B_r \gg B_z$, a common assumption for AF-MPDTs.

Since the AF-MPDT under consideration follows cylindrical geometry, the *r*-component of the momentum equation is given by


(29) \begin{eqnarray*}
&&\rho \bigg ( v_r \displaystyle\frac{\partial v_r}{\partial r} + \displaystyle\frac{v_\theta }{r} \displaystyle\frac{\partial v_r}{\partial \theta } + v_z \displaystyle\frac{\partial v_r}{\partial z} - \displaystyle\frac{v_\theta ^2}{r} \bigg ) \\
&&\qquad = -\displaystyle\frac{\partial p}{\partial r} + \displaystyle\frac{1}{r} \displaystyle\frac{\partial }{\partial r} ( r \eta _{rr} ) + \displaystyle\frac{1}{r} \displaystyle\frac{\partial \eta _{r\theta }}{\partial \theta } \\
&& \quad\qquad - \displaystyle\frac{\eta _{\theta \theta }}{r} + \frac{\partial \eta _{rz}}{\partial z} + J_\theta B_z.
\end{eqnarray*}

The $\theta$-component of the momentum equation can be simplified as


(30) \begin{eqnarray*}
&&\rho \bigg ( v_r \frac{\partial v_\theta }{\partial r} + \frac{v_\theta }{r} \frac{\partial v_\theta }{\partial \theta } + v_z \frac{\partial v_\theta }{\partial z} + \frac{v_r v_\theta }{r} \bigg ) \\
&&\qquad = -\frac{1}{r} \frac{\partial p}{\partial \theta } - \frac{1}{r^2} \frac{\partial }{\partial r} ( r^2 \eta _{r\theta } ) + \frac{1}{r} \frac{\partial \eta _{\theta \theta }}{\partial \theta } \\
&&\qquad \quad + \frac{\partial \eta _{\theta z}}{\partial z} + J_z B_r - J_r B_z.
\end{eqnarray*}

The *z*-component of the momentum equation is


(31) \begin{eqnarray*}
&&\rho \bigg ( v_r \frac{\partial v_z}{\partial r} + \frac{v_\theta }{r} \frac{\partial v_z}{\partial \theta } + v_z \frac{\partial v_z}{\partial z} \bigg ) \\
&&\qquad = -\frac{\partial p}{\partial z} - \frac{1}{r} \frac{\partial }{\partial r}(r \eta _{rz}) \\
&&\qquad \quad +\, \frac{1}{r} \frac{\partial \eta _{\theta z}}{\partial \theta } + \frac{\partial \eta _{zz}}{\partial z} - J_\theta B_r.
\end{eqnarray*}

Here, $v_i$, $i = r, \theta , z$ are the radial, azimuthal and axial plasma velocities, $\rho$ is the plasma density and *p* is the pressure. The terms $\eta _{rr}, \eta _{rz}, \eta _{r\theta }, \eta _{\theta \theta }, \eta _{\theta z}, \eta _{zz}$ are the components of the viscosity tensor. The terms $B_i,\, i = r, \theta , z$, and $J_i,\, i = r, \theta , z$ are the components of the magnetic field and plasma current density in the radial, azimuthal and axial directions, respectively.

In the expansion region, the dominant magnetic field lines typically follow the radial direction, creating a magnetic nozzle-like geometry. This configuration helps to channel and accelerate the plasma flow in the axial direction, which is essential for thrust generation in MPDTs, as the plasma must detach from the field lines to produce thrust.

By inserting these stress terms and performing mathematical manipulation, we obtained a simplified form of the axial velocity of the plasma from Equation (1).

## Experimental method

The experiment was conducted using a 25 kW HTS AF-MPDT, downscaled to 10–15 kW. Details regarding the target-based thrust measurement follow the methodology delineated in [13]. Figure 4 presents the comprehensive schematic of the experimental setup. The system consists of a hollow tungsten cathode, copper anode, HTS magnet and a thrust target positioned 35 cm downstream of the thruster exit. The entire assembly is housed within a vacuum chamber. The HTS magnet is actively cooled via a Stirling-cycle conduction-cooling system. Power to the cathode, anode and magnet is supplied by independent power supplies. An ultrahigh-speed camera was employed to capture time-resolved images of the plasma plume. Argon gas, introduced from external cylinders, was supplied at mass flow rates of 5, 20 and 40 mg/s. Upon ignition, the cathode emits electrons, initiating plasma formation. The plasma is then accelerated by the magnetic nozzle-like field topology generated by the HTS magnet, which produces a uniform and gradually diverging channel. This configuration directs the plasma flow toward the thrust target located 35 cm downstream, enabling direct thrust measurement.

## Langmuir probe diagnostics

To initiate the diagnostic process, a single Langmuir probe, mounted on a movable stage and referred to as ‘Hidden ES-Pion’, sweeps the potential from $-30$ V to 30 V [38–40]. Current-voltage (*I*-*V*) characteristics were recorded systematically at various radial and axial positions. These data, captured via a movable probe, enabled the extraction of key plasma parameters, including electron density, ion density and ion flux.

## Supplementary Material

nwaf589_Supplemental_File

## References

[1] Levchenko I, Xu S, Teel G et al. Recent progress and perspectives of space electric propulsion systems based on smart nanomaterials. Nat Commun 2018; 9: 879. 10.1038/s41467-017-02269-7 29491411 PMC5830404

[2] Rafalskyi D, Martínez JM, Habl L et al. In-orbit demonstration of an iodine electric propulsion system. Nature 2021; 599: 411–5. 10.1038/s41586-021-04015-y 34789903 PMC8599014

[3] De Bruin J, Camino O, Bodin P et al. SMART-1 lunar mission: from capture to impact [abstract]. Presented at the 57th International Astronautical Congress, Valencia, Spain, 2–6 October 2006.

[4] Russell CT, Raymond CA. The dawn mission to Vesta and Ceres. Space Sci Rev 2011; 163: 3–23. 10.1007/s11214-011-9836-2

[5] Nishiyama K, Hosoda S, Tsukizaki R et al. In-flight operation of the Hayabusa2 ion engine system on its way to rendezvous with asteroid 162173 Ryugu. Acta Astronaut 2020; 166: 69–77. 10.1016/j.actaastro.2019.10.005

[6] Tsuda Y, Yoshikawa M, Hirabayashi M et al. Hayabusa2 as the beginning of deep space sample return. In: Hirabayashi M, Tsuda Y (eds). Hayabusa2 Asteroid Sample Return Mission. Amsterdam: Elsevier, 2022, 1–3.

[7] Coogan W, Choueiri E. A critical review of thrust models for applied-field magnetoplasmadynamic thrusters. In: 53rd AIAA/SAE/ASEE Joint Propulsion Conference. Reston, VA, 2017. Abstract 1–27.

[8] Zhang G, Ren J, Liu Q et al. Development of a low-power Hall thruster with permanent magnets and a dual trigger electrode hollow cathode for the Qilu satellite constellation. Aerosp Sci Technol 2024; 154: 109538. 10.1016/j.ast.2024.109538

[9] Zheng J, Liu H, Song Y et al. Integrated study on the comprehensive magnetic-field configuration performance in the 150 kW superconducting magnetoplasmadynamic thruster. Sci Rep 2021; 11: 20706. 10.1038/s41598-021-00308-4 34667219 PMC8526827

[10] Boxberger A, Herdrich G. Integral measurements of 100 kW class steady state applied-field magnetoplasmadynamic thruster SX3 and perspectives of AF-MPD technology [abstract]. Presented at the 35th International Electric Propulsion Conference, Atlanta, Georgia, 8–12 October 2017.

[11] Aglietti G, Redi S, Tatnall A et al. Aerostat for solar power generation. Solar Energy InTech 2010; 399–412.

[12] Acheson CR, Kinefuchi K, Ichihara D et al. Operation of a plasma thruster featuring a 1.1 T high temperature superconducting magnet. J Electr Propuls 2024; 3: 17. 10.1007/s44205-024-00080-3

[13] Aftab H, Jinxing Z, Liu H et al. Exploring efficiency in next generation high temperature superconducting-enhanced applied field magnetoplasmadynamic thrusters: a combined numerical and experimental study. Acta Astronaut 2024; 223: 448–61. 10.1016/j.actaastro.2024.07.033

[14] Voronov A, Troitskiy A, Egorov I et al. Magnetoplasmadynamic thruster with an applied field based on the second generation high-temperature superconductors. J Phys: Conf Ser 2020; 1686: 012023. 10.1088/1742-6596/1686/1/012023

[15] Winter M, Herdrich G, Bögel E et al. Downscaling the 100 kW SX-3 AF-MPD to the 5 kW SUPREME thruster. J Electr Propuls 2025; 4: 1–32. 10.1007/s44205-025-00128-y

[16] Maeshima D, Mori R, Inoue S et al. MPD thruster with a strong applied field up to 0.5 T using cryogenic fluid-cooled magnetic coil. J Electr Propuls 2025; 4: 1–12. 10.1007/s44205-025-00110-8

[17] Bögel E, Collier-Wright M, Aggarwal K et al. State of the art review in superconductor-based applied-field magnetoplasmadynamic thruster technology [abstract]. Presented at the 37th International Electric Propulsion Conference, Cambridge, MA, 19–23 June 2022.

[18] Kinefuchi K, Wimbush S, Ichihara D et al. Performance evaluation of a plasma thruster using a high-temperature superconducting magnet. Trans JSASS Aerospace Tech Japan 2024; 22: aj1–6.

[19] Collier-Wright M, Bögel E, Betancourt MLR et al. High-temperature superconductor-based power and propulsion system architectures as enablers for high power missions. Acta Astronaut 2022; 201: 198–208. 10.1016/j.actaastro.2022.08.035

[20] Liu H, Zheng J, Zhu X et al. A miniaturized conduction-cooled hts magnet for space magnetoelectric thruster. IEEE Trans Appl Supercond 2022; 33: 1–8.

[21] Rey C, Hoffman W, Chang-Diaz F et al. Design and fabrication of an HTS magnet for the VASIMR experiment. IEEE Trans Appl Supercond 2002; 12: 993–6. 10.1109/TASC.2002.1018567

[22] Shao K, Gu D, Ju B et al. Analysis of Tiangong-2 orbit determination and prediction using onboard dual-frequency GNSS data. GPS Solut 2020; 24: 11. 10.1007/s10291-019-0927-y

[23] Sasoh A, Arakawa Y. Thrust formula for applied-field magnetoplasmadynamic thrusters derived from energy conservation equation. J Propuls Power 1995; 11: 351–6. 10.2514/3.51432

[24] Tobari H, Ando A, Inutake M et al. Characteristics of electromagnetically accelerated plasma flow in an externally applied magnetic field. Phys Plasmas 2007; 14: 093507. 10.1063/1.2773701

[25] Fradkin D, Blackstock A, Roehling D et al. Experiments using a 25-kW hollow cathode lithium vapor MPD arcjet. AIAA J 1970; 8: 886–94. 10.2514/3.5783

[26] Tikhonov V, Semenikhin S, Alexandrov V et al. Plasma acceleration in self-field and AF-MPD thrusters [abstract]. Presented at the Proceedings of the 23rd International Electric Propulsion Conference, Seattle, WA, 13–16 September 1993.

[27] Herdrich G, Boxberger A, Petkow D et al. Advanced scaling model for simplified thrust and power scaling of an applied-field magnetoplasmadynamic thruster. In: 46th AIAA/ASME/SAE/ASEE Joint Propulsion Conference & Exhibit. Reston, VA, 2010. Abstract doi: 10.2514/6.2010-6531. 10.2514/6.2010-6531

[28] National High Magnetic Field Laboratory. Engineering Critical Current Density vs. Applied Field for superconductors available in long lengths. 2018. https://nationalmaglab.org/media/4v0fnuzu/je_vs_b-041118a.pdf (April 2018).

[29] MYERS R . Scaling of 100 kW class applied-field MPD thrusters [abstract]. Presented at the 28th Joint Propulsion Conference and Exhibit, Nashville, TN, 6–8 July 1992.

[30] Kitaeva A, Tang H, Wang B et al. Theoretical and experimental investigation of low-power af-mpdt performance in the high mass flow rate low discharge current regime. Vacuum 2019; 159: 324–34. 10.1016/j.vacuum.2018.10.046

[31] Kasuga H, Jeong J, Mizutani K et al. Operation characteristics of applied-field magnetoplasmadynamics thruster using hollow cathode. Trans JSASS Aerospace Tech Japan 2018; 16: 69–74.

[32] Nabuchi H, Suzuki K, Kobayashi Y et al. Thrust enhanced by a magnetic laval nozzle in an applied-field magnetoplasmadynamic thruster. Plasma Fusion Res 2016; 11: 2406033. 10.1585/pfr.11.2406033

[33] Nakano T, Ishiyama A, SHIMIZU Y et al. Feasibility study of a low-power applied-field MPD arcjet [abstract]. Presented at the 28th International Electric Propulsion Conference, Toulouse, France, 17–21 March 2003.

[34] Kawasaki A, Kubota K, Funaki I et al. MHD simulation and thermal design of an MPD thruster. Trans JSASS Aerospace Tech Japan 2014; 12: Pb_19–25.

[35] Haag D, Fertig M, Auweter-Kurtz M. Numerical simulations of magnetoplasmadynamic thrusters with coaxial applied magnetic field [abstract]. Presented at the Proceedings of the 30th International Electric Propulsion Conference, Florence, Italy, 17–20 September 2007.

[36] Lev D . Investigation of efficiency in applied field magnetoplasmadynamic thrusters. Ph.D. Thesis. Princeton University, Department of Mechanical and Aerospace Engineering, 2012.

[37] Albertoni R . Cathode processes in MPD thrusters. *Ph. D. Thesis*. Università degli Studi di Pisa, Department of Civil and Industrial Engineering, 2012.

[38] Chai KB, Shin C, Yoon YD et al. Observation of fast Alfvén wave in KAERI plasma beam irradiation facility using an applied-field MPD thruster. Phys Plasmas 2024; 31: 043513. 10.1063/5.0198223

[39] Xia Y, Yang X, Chang L et al. Development of a compact helicon plasma source with two sets of ring array permanent magnets for the study of blue core plasma. Rev Sci Instrum 2023; 94: 125110. 10.1063/5.0170135 38126814

[40] Rohkamm E, Spemann D, Scholze F et al. Characterization of an RF excited broad beam ion source operating with inert gases. J Appl Phys 2021; 129: 223305. 10.1063/5.0052758

