# Supplementary material for: High performance of high-temperature-superconducting MPD thrusters: analytical MHD modeling and experimental demonstration
Source: Natl Sci Rev. 2025 Dec 30;13(2):nwaf589. doi: 10.1093/nsr/nwaf589 (PMC12839541; doi:10.1093/nsr/nwaf589)
Supplement: nwaf589_Supplemental_File [file nwaf589_supplemental_file.docx]

Supplementary data for

**High performance in high-temperature superconducting MPD thrusters: Analytical MHD modeling and experimental demonstration**

Jinxing Zheng^1*,+^, Yifan Du^1,2,+^, Hammad Aftab^1,2,+^, Haiyang Liu^1+^, Ming Li^1^, Zhu Lei^1^, Yudong Lu^1^, Maolin Ke^1,2^, Ming Zhu^1^, Juan Wu^1,2^, and Bofan Li^,2^

1 Institute of Plasma Physics, Hefei Institutes of Physical Sciences Chinese Academy of Sciences, Hefei 230031, China

2 University of Science and Technology of China, Hefei, 230026, China

**Author to whom any correspondence should be addressed: jxzheng@ipp.ac.cn

**This PDF file includes:**

Supplementary Text

Figs. S1,S5

The file contains the magnetic field topology of the HTS magnet, ad depicted in Figure.S1.


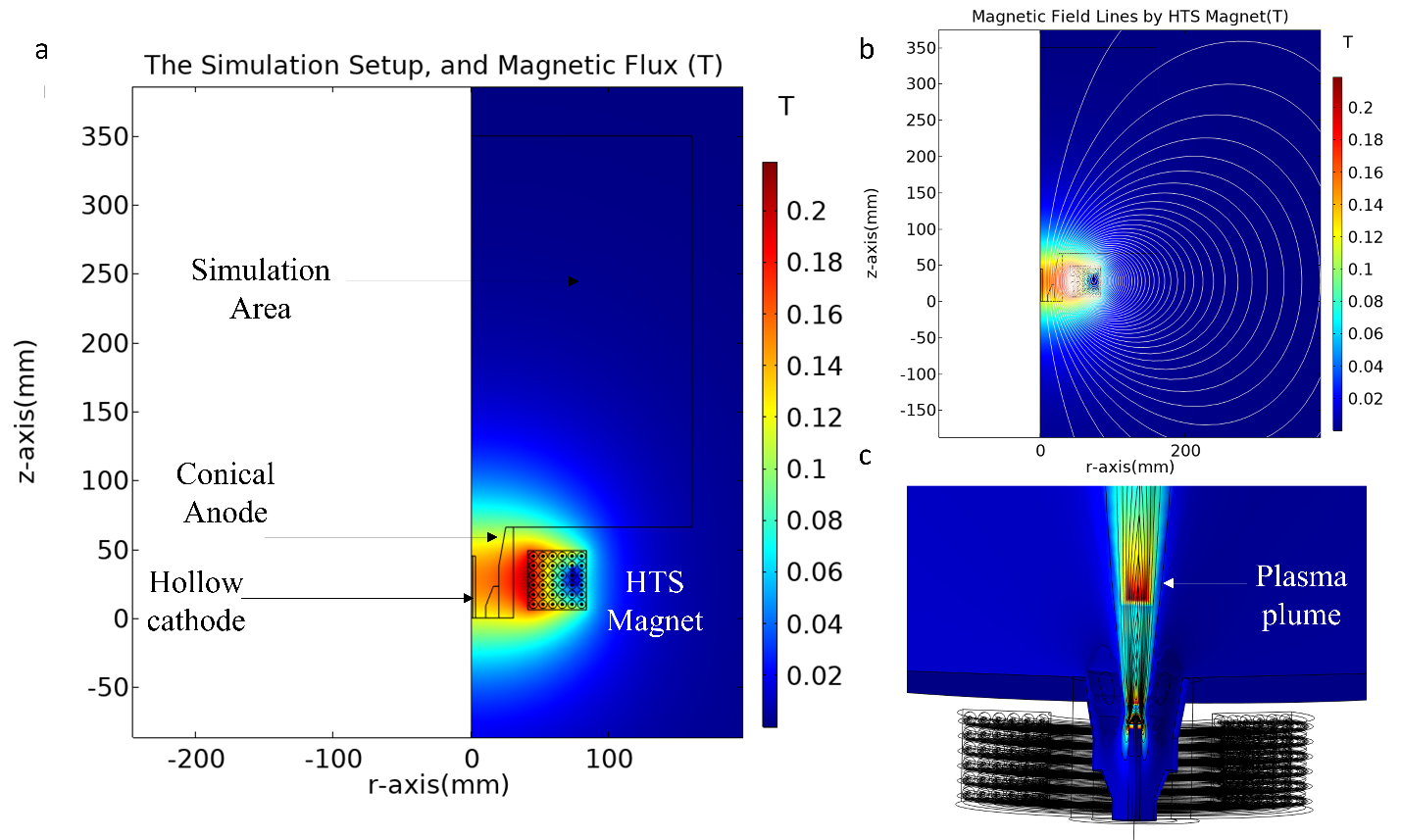


**Figure S1**: Numerical Setup and High-Temperature Superconducting (HTS) Magnet:
(a) Numerical Model Featuring an HTS Magnet Producing a 0.2 T Magnetic Field; (b)
Magnetic Field Line Topology of the HTS Magnet; (c) Three-Dimensional View of the
Setup with Plasma Plume

**Figure S1(a)** shows the simulated configuration of the HTS magnet and its magnetic field distribution, reaching strengths up to 0.2 T. **Figures S1(b–c)** illustrate the central magnetic field profile, also reaching up to 0.2 T. The magnet dimensions are 40 mm in width and 41 mm in length.

**
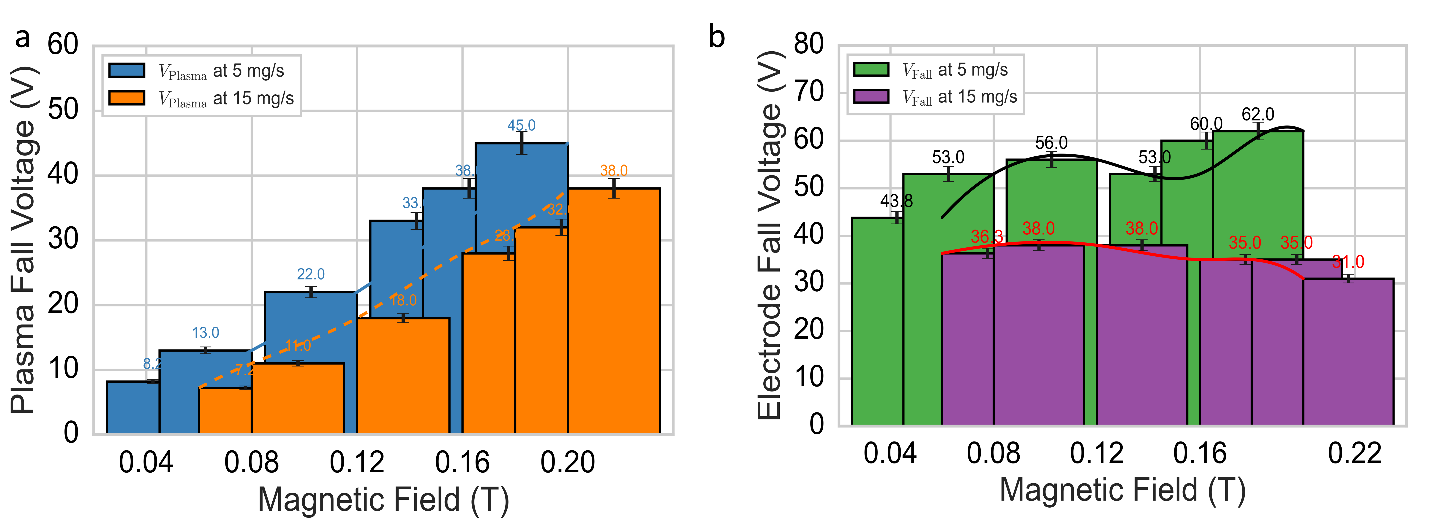
**

**FigureS2:** (a) Plasma fall voltage vs magnetic field strength. (b) Electrode fall voltage vs magnetic field at 5mg/sand15mg/s in the experimental setup.

**Figure S2(a–b)** shows the variation of plasma fall voltage and electrode fall voltage with applied magnetic field strength. As the magnetic field increases, the plasma absorbs more voltage (or power), whereas the voltage lost to the electrodes does not exhibit a consistent trend.

# **Analytical Mode Missing Steps:**

Since the derivation involves large equations, some of them are displayed here. The constants obtained from the manipulation of equations are as follows:

$A=\frac{v_{0}-\frac{R_{0}^{2}-R_{i}^{2}}{6\mu}\frac{\partial}{\partial z}\left( p+\frac{B_{r}^{2}}{\mu_{0}} \right)}{\ln\left( \frac{R_{0}}{R_{i}} \right)}$ (A1)

$B=v_{0}\left( 1-\ln\left( R_{0} \right) \right)+\frac{R_{0}^{2}\ln\left( R_{i} \right)-R_{i}^{2}\ln\left( R_{0} \right)}{6\mu\ln\left( \frac{R_{i}}{R_{0}} \right)}\frac{\partial}{\partial z}\left( p+\frac{B_{r}^{2}}{\mu_{0}} \right)$ (A2)

The swirl component of thrust can be written as:

$F_{\text{swirl}}=\frac{2\pi}{\mu_{0}\sqrt{2\rho}}\int_{0}^{L_{M}} \int_{0}^{R_{0}} \left[ \frac{1}{r^{2}}\frac{\partial}{\partial r}\left( r\frac{\partial}{\partial r}\left( \frac{1}{r}\left( r\frac{\partial}{\partial z}\left( \sqrt{p+\frac{B_{r}^{2}}{\mu_{0}}} \right) \right) \right) \right) \right]dzdr$ (A3)

The$k_{1}$ and$k_{2}$ are defined as:

$k_{1}=\frac{R_{i}^{2}}{6\mu}-\frac{R_{0}^{2}-R_{i}^{2}}{6\mu\ln\left( \frac{R_{i}}{R_{0}} \right)}\ln\left( R_{i} \right)+\frac{R_{0}^{2}\ln\left( R_{i} \right)-R_{i}^{2}\ln\left( R_{0} \right)}{6\mu\ln\left( \frac{R_{i}}{R_{0}} \right)}$ (A4)

And,

$k_{2}=1-\ln\left( \frac{r}{R_{0}} \right)$ (A5)

Finally, $k_{3}$ can be written as:

$k_{3}=\frac{r^{2}}{6\mu}-\frac{R_{0}^{2}-R_{i}^{2}}{6\mu\ln\left( \frac{R_{i}}{R_{0}} \right)}\ln\left( r \right)+\frac{R_{0}^{2}\ln\left( R_{i} \right)-R_{i}^{2}\ln\left( R_{0} \right)}{6\mu\ln\left( \frac{R_{i}}{R_{0}} \right)}$ (A6)

The final thrust expression for HTS-AFMPD is given by:

$F_{\text{total}}=\frac{\mu_{0}^{\frac{1}{2}}\dot{m}k_{3}L_{c}^{2}CI_{d}^{\frac{2}{3}}}{\left( \left( 2\pi r_{\text{exit}} \right)^{\frac{2}{3}}\left( R_{\text{inner}}+R_{\text{outer}} \right) \right)^{2}R_{\text{in}}\sqrt{B_{\text{th}}^{\frac{2}{3}}-\left( \frac{u_{0}I_{d}}{2\pi r_{\text{exit}}} \right)^{\frac{2}{3}}}}B_{\text{th}}^{2}+\left[ F_{Hall} k_{2}+\frac{L_{c}k_{4}C}{\mu_{0}^{\frac{2}{3}}L_{m}\left( R_{\text{inner}}+R_{\text{outer}} \right)} \right]B_{\text{th}}$ (A7)

This relation is analyzed for different flow rates and magnetic fields for comparison with experimental thrust.

## **Experimental target thrust measurement method:**

The thrust measurement system measures the thrust by calibrating the elasticity coefficient of the flexure beam. The target is placed in the plasma plume generated by the electric thruster, and fixedly connected to the flexure beam through the clamp. The momentum of the plasma plume transferred to the target surface is converted into the force applied to the target, which manifests itself as the deformation of the flexure beam in the horizontal direction. The deflection value is measured by the laser displacement sensor, and the thrust force generated by the electric thruster is calculated then.

The moving platform in **Figure.S3** can realize axial movement within a range of 1,800 mm, allowing it to measure the thrust values generated by the electric thrusters in multiple positions and under different operating conditions. The measurement system meets the demand of thrust measurement from 50mN to 10 N, and the laser displacement sensor used to measure the deflection value has a resolution of 0.25 µm, and the measurement error of the thrust measurement platform is 2mN under ideal condition.

The thrust measurement method based on the flexure beam structure is an indirect measurement method, which utilizes the target to intercept the plume generated by the thruster, and calculates the momentum transferred to the target surface by observing the deformation amount of the flexure beam in the horizontal direction, allowing for accurate measurement of the thrust generated by the thruster.

Firstly, the internal mechanics of the thruster is analyzed, with the axial direction of the thruster as the z-axis, and the leftward direction as positive. The thrust generated by the thruster is denoted as $F$; the force of the thruster on the propellant is $F^{'}$; the relationship between $F$ and $F^{'}$ can be obtained:

$F=F^{'}-P_{0}A_{e}=\int_{A_{e}} \rho v_{ez}\left( v_{e}\cdotⅆA \right)+(P_{e}-P_{0})A_{e}$ (A8)

where $P_{0}$ is the atmospheric pressure, $A_{e}$ is the area of the thruster outlet, $v_{e}$ is the exhaust velocity of the propellant after ionization to plasma, $v_{ez}$ is the axial component of $v_{e}$, and $P_{e}$ is the pressure at the thruster outlet.

The momentum equation in the z-axis direction can then be obtained by considering the control volume $\Omega$ on the right-hand side of **Figure S3**:

$T^{'}-P_{e}A_{e}-P_{0}\left( A_{T}-A_{e} \right)=\int_{A_{e}} \rho v_{ez}\left( v_{e}\cdotⅆA \right)$ (A9)

Where $T^{'}$ is the force of the target on the plasma plume and $A_{T}$ is the area of the target. Then, the support force $T$ of the flexural beam on the target can be expressed as:

$T=T^{'}-P_{0}A_{T}$ (A10)

Substituting **Eq. (A9)** into **Eq. (A10)** yields:

$T=\int_{A_{e}} \rho v_{ez}\left( v_{e}\cdotⅆA \right)+(P_{e}-P_{0})A_{e}$ (A11)

That is, the thrust force $F$ generated by the thruster is numerically equal to the support force $T$ of the flexure beam on the target, which theoretically proves the accuracy of the measurement platform of the flexure beam structure.

Therefore, the problem transforms from “how to measure the thrust force $F$ generated by the electric thruster” to “how to measure the support force $T$ of the flexure beam on the target”. In the flexible beam structure of the thrust measurement platform, the flexible beam has a rectangular cross-section, and its length is much greater than its cross-section size, classifying it as a slender beam in the structural mechanics. It can be analyzed using the Euler Bernoulli beam theory. Let the force of the target on the flexural beam be $-T$, and the deflection of the flexural beam due to the flexural behavior be $s$, which can be obtained:

$s=\frac{4l^{3}}{Ebh^{3}}T$ (A12)

where $E$ is the Young's modulus of the flexural beam, and $l$, $b$ and $h$ are the length, width and thickness of the flexural beam, respectively. Assuming that the scaling factor between the deflection $s$ and the output signal $\Delta V$ of the laser displacement sensor is $k_{1}$, which is obtained when brought into **Eq. (A5)**:

$T=\frac{Ebh^{3}}{4l^{3}}k_{1}\Delta V$ (A13)

Let$Ebh^{3}/4l^{3}\cdot k_{1}=k$ , and we get:

$T=k\Delta V$ (A14)


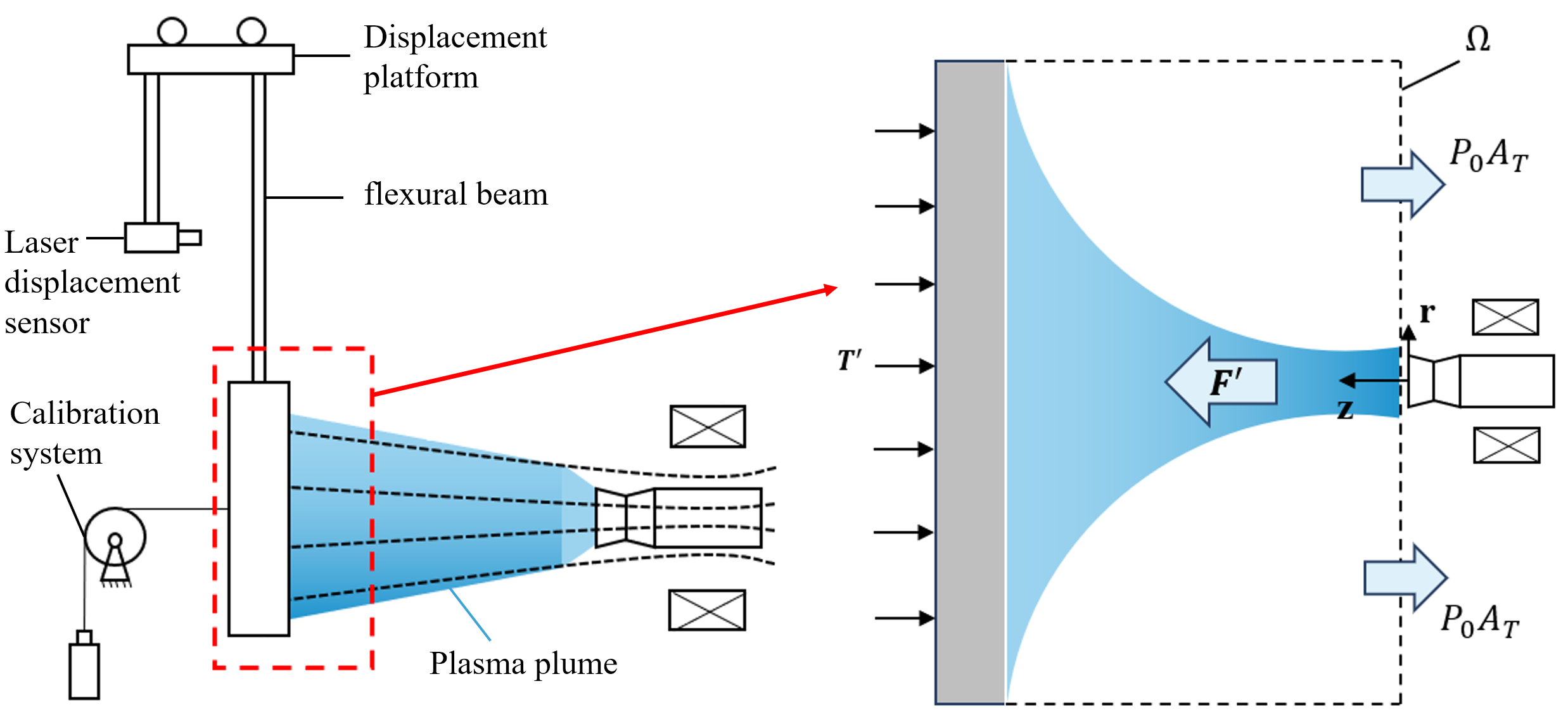


Figure S3: Principle diagram of thrust measurement system.

Where $k$ can be considered as the elasticity coefficient of the thrust measurement platform (flexure beam), and its specific value can be obtained through the calibration system. The proportionality coefficient $k$ between the deflection of the flexure beam and the standard force is calculated by calibrating the device before the experiment. During the experiment, the thrust produced by the thruster can be calculated by observing and recording the deflection of the flexure beam.


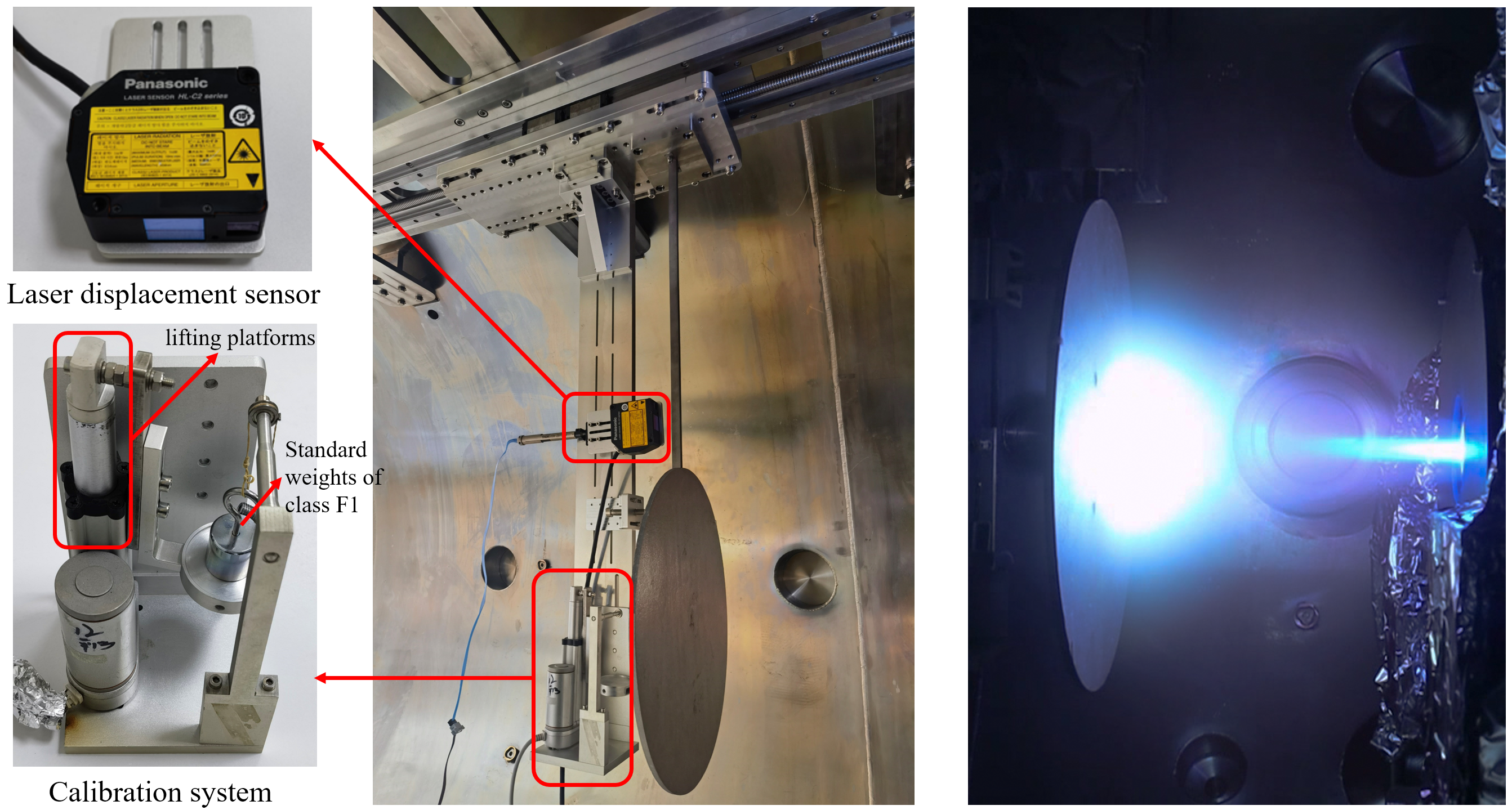


Figure S4: Illustration of thrust measurement system

**Figure S4** shows the platform of thrust measurement system, primarily comprising the displacement measurement system, measurement and control system and calibration system. Both the displacement measurement system and calibration system are housed within the vacuum equipment. Remote completion of calibration experiments outside the vacuum equipment is facilitated through the calibration system. The displacement measurement system employs a laser displacement sensor for the signal acquisition. The distance between the sensor and the deflection beam can be adjusted through the small displacement controller to ensure it remains within the sensor's measurement center distance.

After accounting for the neutral beam rebound effect and other inaccuracies, the final relation for thrust calculation in the experimental setup is given as follows:

$F_{M}=k_{1}T-3.07\dot{m}\sqrt{\frac{T_{k}}{M}}$ (A15)

$\dot{m}$ is mass flow rate, $k_{1}$ is coefficient of beam, where $T_{K}$ is the surface temperature of the target and $M$ is the molar mass of the gaseous propellant molecules.

# **Power Analyses**

In **Figure 4** of the main text, the results correspond to the testing of the HTS magnet alone, when it was not integrated into the Applied-Field MPD thruster system. However, during the experiments where the magnet was integrated into the thruster, the operating current was limited to 25 A to ensure safe operation. This precaution was necessary because HTS tapes are costly and must be kept within their safe current range to prevent damage.

Also as depicted in **Figure.S5,** the power in the experimental setup varies as a function of magnetic field. At a mass flow rate of 20~mg/s, the power increases almost linearly with magnetic field, whereas at 40mg/s, the power decreases at higher magnetic field values. It is evident that lower mass flow rates correspond to higher injected power, while higher mass flow rates result in lower injected power. This explains why, in many thruster designs, increasing the mass flow rate enhances both thrust and overall performance. Overall, higher magnetic fields lead to increased power at 5mg/s and 20mg/s, whereas at 40mg/s, the trend is different, showing a decreasing behavior at higher fields. Thus the power injected play a significant role in thrust improvement.


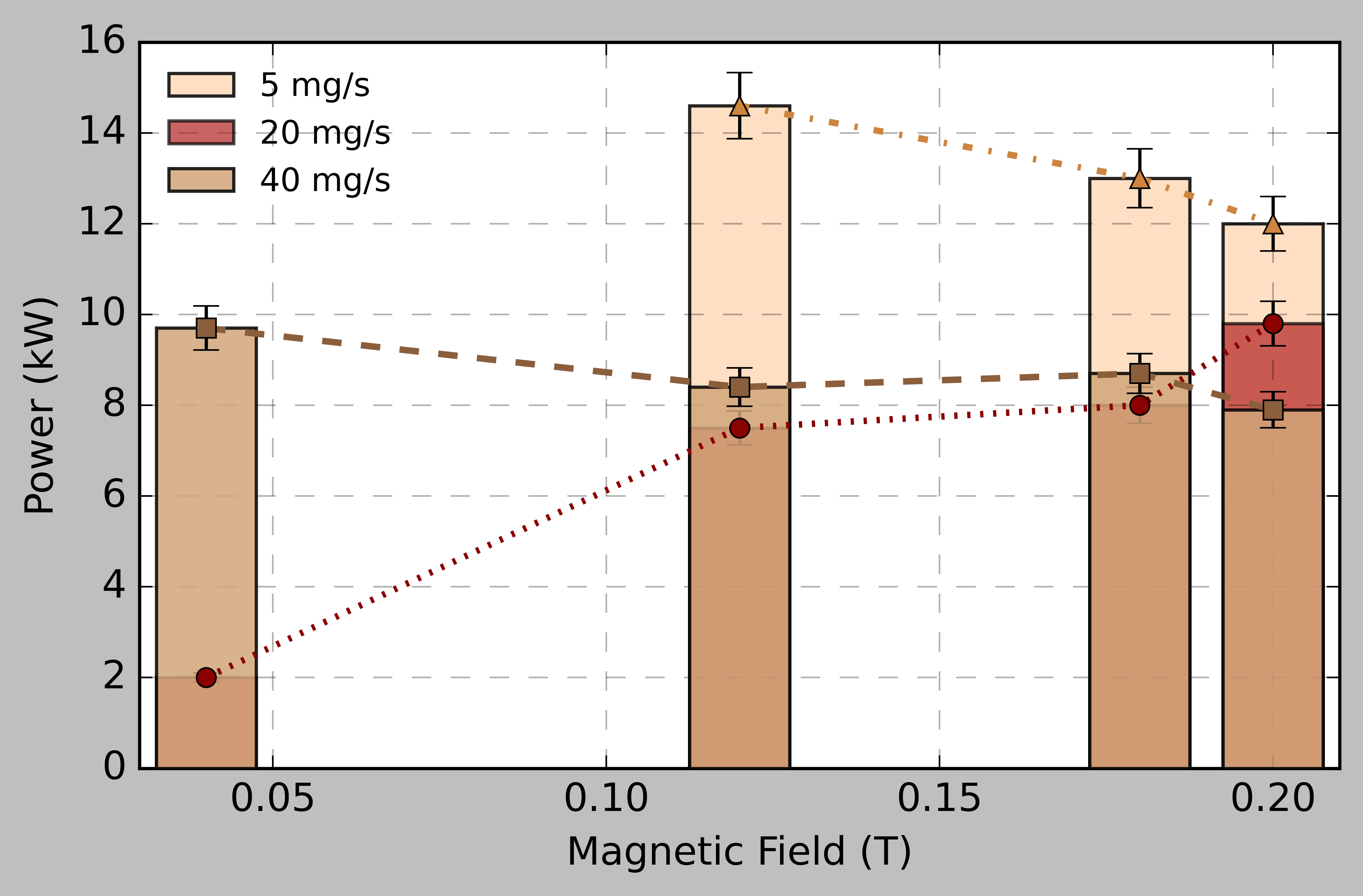


**Figure S5**: The variation of power with magnetic field strength in the experimental setup for 5mg/s, 20mg/s and 40mg/s
